# Red Ghost Image Elimination Method Based on Driving Waveform Design in Three-Color Electrophoretic Displays

**DOI:** 10.3390/mi13020275

**Published:** 2022-02-08

**Authors:** Li Wang, Wenjun Zeng, Zhuopei Liang, Guofu Zhou

**Affiliations:** 1 School of Information Engineering, Zhongshan Polytechnic, Zhongshan 528400, China; creekxi@163.com; 2 Guangdong Provincial Key Laboratory of Optical Information Materials and Technology & Institute of Electronic Paper Displays, South China Academy of Advanced Optoelectronics, South China Normal University, Guangzhou 510006, China; guofu.zhou@m.scnu.edu.cn; 3 College of Electron and Information, University of Electronic Science and Technology of China, Zhongshan Institute, Zhongshan 528402, China; Liang88320733@163.com

**Keywords:** three-color electrophoretic displays, driving waveform, red ghost images, microcapsule technology, flickers

## Abstract

Three-color electrophoretic displays (EPDs) are a new type of optoelectronic display device. However, they have the defect of red ghost images during gray scale transformation, which affects the accuracy of the gray scale display. In this paper, we proposed a new driving method for eliminating the red ghost images. A driving waveform was composed of an erasing stage, an activation stage, and a driving stage. First, the erasing stage was subdivided into a red erasing stage and an original erasing stage, the red erasing stage was used to eliminate residual red particles in the top of the microcapsules. Then, a high-frequency square wave was used as the activation stage for increasing the activity of the black and white particles. Meanwhile, the intensity of flickers could be decreased by the high-frequency square wave. Finally, the performance of the driving waveform was tested by a colorimeter. The experimental results showed that the driving waveform could effectively eliminate red ghost images by 80.43% and reduce the flicker intensity by 79.63%, compared with an existing driving waveform.

## 1. Introduction

Electronic paper is an ultra-low energy consumption optoelectronic display technology [1,2,3,4]; it mainly includes electrowetting displays (EWDs) [5,6,7] and electrophoretic displays (EPDs) [8,9]. Types of EPDs include traditional black/white EPDs [10] and three-color EPDs [11]. Between them, the three-color EPD is an improved display technology based on new red electrophoretic particles in microcapsules, it could effectively compensate for the limitations of traditional EPDs in color display [12]. However, some new problems have arisen in three-color EPDs. For example, red particles cannot be sufficiently eliminated when they are driven to the top of microcapsules, which leads to the appearance of red ghost images in a gray scale transformation. A voltage sequence used to control the gray scale transformation is defined as a driving waveform, and particle motion is controlled by it [13]. However, the design of traditional driving waveforms cannot effectively eliminate red ghost images, which reduce the accuracy of the gray scale display [14]. Therefore, it is of great significance to eliminate the red ghost images by optimizing the driving waveforms of three-color EPDs.

The problem of ghost images has been discussed in traditional EPDs [15]. The reason for ghost images is that an original image remains in a new image display. The process of image display is completed by controlling the movement of particles, which depends on an electric field generated by driving waveforms [16]. A physical model of the electric field has been proposed, which provides theoretical guidance for driving waveform design [17]. Traditional driving waveforms include an erasing stage, an activation stage, and a driving stage [18]. The erasing stage is used to erase the original images, and its design plays a key role in eliminating ghost images. So, a driving method based on the optimization of an erasing stage was proposed to eliminate ghost images [19]; a time parameter was added in the erasing stage to compensate for the response delay. However, the driving waveform did not follow the direct current (DC) balance rule because of this added time parameter, which could damage three-color EPDs [20]. The activation stage is applied after the end of the erasing stage; its function is to improve the activity of particles, which has an important influence on the elimination of ghost images. The design of the activation stage generally is a single square wave or multiple square waves [21]. For example, a square wave with an inflection point was used as the activation stage to improve the activity of particles [22]. However, the period of the square wave was too long, which could cause ghost images. Ghost images can also be suppressed by optimizing the gray scale transformation algorithms, therefore, an edge detection algorithm was used to improve the accuracy of the gray scale display [23]. In addition, an intelligent software system design is helpful to eliminate ghost images, so a convolutional neural network was used to intelligently suppress the ghost images in EPDs [24]. Finally, the improvement of electrophoretic particle properties can effectively improve the display quality of EPDs [25,26]. However, the existing driving methods for three-color EPDs do not consider the influence of the red particles on the gray scale display, and the problem of red ghost images has not been effectively solved.

In this paper, a new driving waveform was proposed for eliminating red ghost images in three-color EPDs. First, the display principle of the three-color EPDs was analyzed by using electrophoresis theory. Then, the movement of red particles in a gray scale transformation was analyzed by using Stokes’ law. Lastly, the red ghost images were eliminated by applying a red erasing stage and a high frequency square wave in the driving waveform.

## 2. System Principle

### 2.1. Display Principle

**Figure 1:**
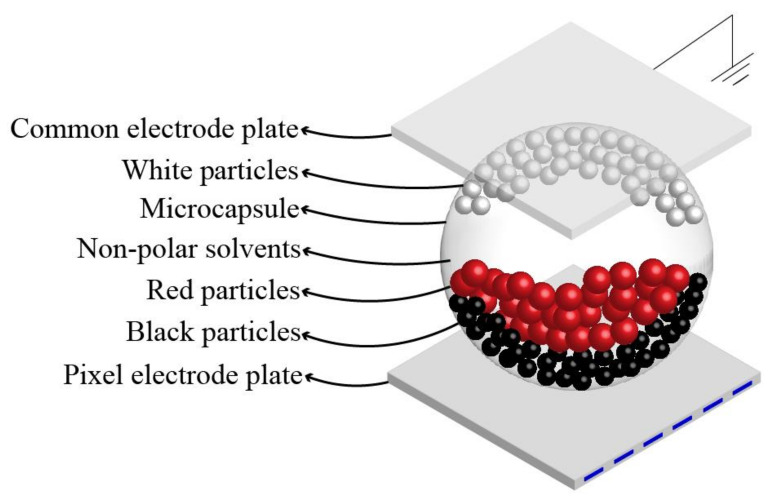
A microcapsule structure of a three-color electrophoretic display (EPD). White particles, black particles, red particles, and nonpolar solvents are encapsulated in a microcapsule. A common electrode plate and a pixel electrode plate are at the top and bottom of the microcapsule. White particles are in the upper part of the microcapsule when a negative driving voltage is applied.

A three-color EPD based on microcapsule technology is shown in Figure 1 [27]. Microcapsule technology is a technology of coating a solid or liquid with film-forming materials. Nonpolar solvents and three types of particles are encapsulated in a microcapsule of three-color EPDs. Among these particles, the charge polarities of the black particles and red particles are positive, but their charge amounts are different, and the charge polarity of white particles is negative. A common electrode plate and a pixel electrode are at the top and bottom of the microcapsule [28,29]. Particles could be driven by applying driving waveforms to two electrode plates. When no driving waveform is applied, the state of the particles could be maintained due to the particle density being equal to the solvent density. So, the pixel could always display the last image: this is a reason for the ultra-low energy consumption of EPDs. In addition, images could be converted by designing driving waveforms based on these principles [30,31].

### 2.2. Red Ghost Images

**Figure 2:**
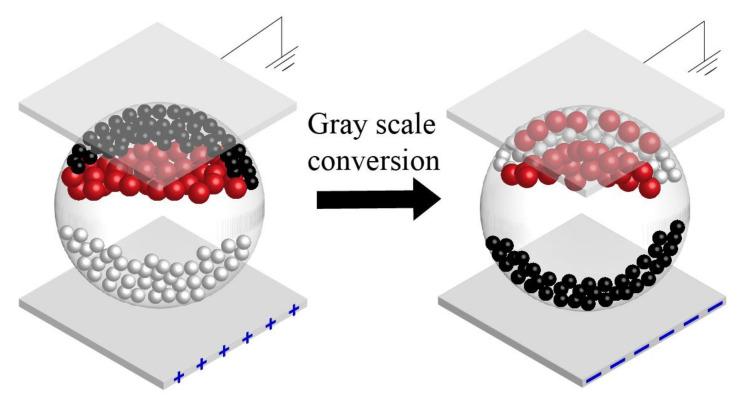
The phenomenon of red ghost images in a gray scale transformation from a black gray scale to a white gray scale. Some red particles are mixed with white particles in the white gray scale.

Red ghost images are a phenomenon in which red particles remain in black or white gray-scale display. The reason for this phenomenon is that red particles could remain in the upper part of microcapsules, and then, they are mixed with other particles. In addition, red ghost images are more obvious in a gray-scale transformation from a black gray to a white gray scale, as shown in Figure 2. The particle distribution of the black gray scale in microcapsules is that the black particles are in the upper part of microcapsules, the red particles are below the black particles, and the white particles are in the lower part of microcapsules. Then, the black particles and red particles could move down when an erasing stage is applied. In this process, the relationship between a resultant force and the velocity of particles could be expressed by Equation (1) [32].

(1) $$Uq/d-6\pi \eta vR=m\left(dv/dt\right)$$

where U is a driving voltage applied between the common and pixel electrode plate, q is charge amount of particles, d is the distance between the common and pixel electrode plate, η is the viscosity coefficient of nonpolar solvents, v is particle velocity, R is particle radius, m is particle mass, the first term is the electric field force generated by the driving voltage, the second term is the viscous force caused by nonpolar solvents, which follows Stokes’ law. The velocity of particles could be obtained by solution of Equation (1), as shown in Equation (2) [33,34].

(2) $$v=\left(Uq/\left(6\pi d\eta R\right)\right)\left(1-e^{-\left(\left(6\pi \eta R\right)/m\right)t}\right)$$

where t is a time variable. It is shown that particle velocity is inversely proportional to its volume. The red particle volume is larger than that of black particles or white particles. Therefore, the black particle velocity is faster than that of red particles. In fact, the driving time of black particles is about hundreds of milliseconds, and the driving time of red particles is about a few seconds. So, red particles could remain in the upper part of microcapsules due to their slow speed. Then, some of red particles are mixed with white particles in the white gray scale when a driving stage is applied. Finally, the white gray scale has red ghost images, and its luminance is decreased. Therefore, red particles need to be effectively controlled by optimizing driving waveforms to eliminate the red ghost images.

### 2.3. Design of Driving Waveforms

**Figure 3:**
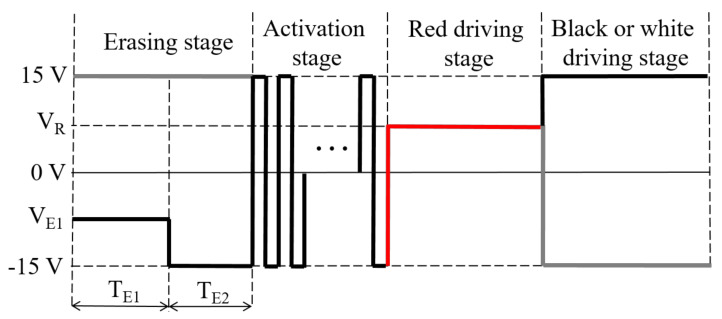
The proposed driving waveform for eliminating red ghost images. The erasing stage is divided into a red erasing stage and an original erasing stage. $V_{R}$ and $V_{E1}$ are the driving voltage of the red driving stage and red erasing stage, respectively, $T_{E1}$ and $T_{E2}$ are the driving time of the red erasing stage and original erasing stage, respectively. The red line represents the red driving stage, the grey line represents the gray scale conversion in the opposite situation.

The design of the erasing stage and the activation stage could easily lead to the occurrence of red ghost images, which affects the display quality of three-color EPDs. Therefore, we proposed a new driving waveform to eliminate red ghost images. The driving waveform includes an erasing stage, an activation stage, a red driving stage, and a black or white driving stage, as shown in Figure 3. The erasing stage is divided into a red erasing stage and an original erasing stage. The red erasing stage is a negative voltage $V_{E1}$, which is used to erase red images. Then, the original erasing stage is used to erase black or white images. The optimization of the erasing stage could increase the moving speed of red particles, which could prevent red particles from mixing with other particles. The activation stage is a high-frequency square wave, and the frequency of the square wave is higher than the frequency range recognized by human eyes. So, the impact of flicker on the visual effect could be reduced. In addition, red particles cannot be driven to the upper part of microcapsules due to their long driving time. Therefore, the activation stage could achieve the purpose of activating particles and avoiding the appearance of red ghost images. The driving stage includes a red driving stage and a black or white driving stage, which are consistent with the traditional driving waveform. Moreover, the proposed driving waveform follows a DC balance rule between an original gray scale and a target gray scale, which could prevent the accumulated charge from damaging three-color EPDs, as shown in Equation (3).

(3) $$V_{E1}T_{E1}+V_{E2}T_{E2}+V_{R}^{’}T_{R}^{’}+V_{D}^{’}T_{D}^{’}=0$$

where the first term and the second term are driving waveform parameters which form the target gray scale, $V_{E1}$ and $T_{E1}$ are the driving voltage and driving time of the red erasing stage, respectively, $V_{E2}$ and $T_{E2}$ are the voltage amplitude and driving time of the original erasing stage, respectively. The third term and the fourth term are driving waveform parameters which form the original gray scale, $V_{R}^{’}$ and $T_{R}^{’}$ are the driving voltage and driving time of the red driving stage, respectively, $V_{D}^{’}$ and $T_{D}^{’}$ are the voltage amplitude and driving time of the black or white driving stage, respectively.

## 3. Experimental Results and Discussion

### 3.1. Experimental Platform

**Figure 4:**
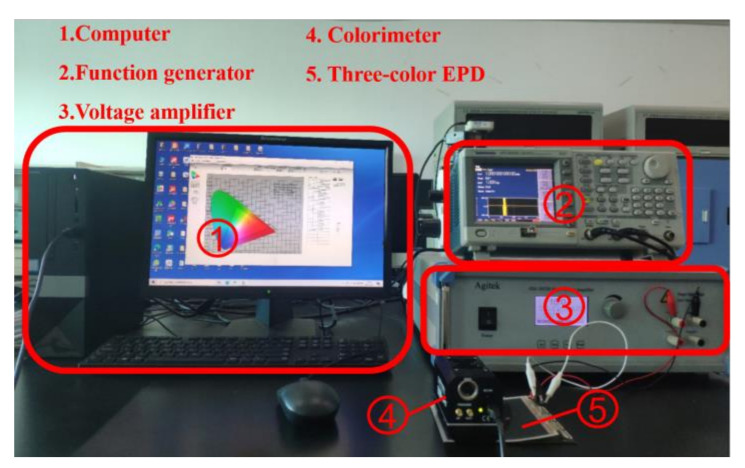
An experimental platform for three-color EPDs. It included a computer, a function generator, a voltage amplifier, a colorimeter, and a three-color EPD. The computer was used to edit driving waveforms, and the function generator and the voltage amplifier were used to generate and output driving waveform data. The colorimeter was used to test and record CIE Yxy data of the three-color EPDs.

The performance of a three-color EPD could be determined by testing against the Commission Internationale de L’Eclairage (CIE) Yxy chromaticity diagram. Y and x are the luminance and a chromaticity coordinate values in the CIE Yxy, respectively. The red saturation is expressed by x, and the luminance is expressed by Y. We established an experimental platform, as shown in Figure 4. First, ArbExpress waveform editing software (V3.4, Tektronix, Beaverton, OR, USA) was used to edit the driving waveforms in a computer (H430, Lenovo, Beijing, China). Then, a function generator (AFG3022C, Tektronix, Beaverton, OR, USA) was used to generate driving waveform data, and a voltage amplifier (ATA-2022H, Agitek, Xian, China) was used to amplify the voltage amplitude of driving waveforms. Next, a three-color EPD (Dalian Longning Technology Co. Ltd., Dalian, China) was connected to the voltage amplifier, and it was driven by driving waveforms. Finally, a colorimeter (Arges-45, Admesy, Ittervoort, The Netherlands) was used to test and record the CIE Yxy data of the three-color EPD. The measurement interval of the colorimeter was 0.11 s, and the chromaticity coordinate of a reference white point was (0.3127, 0.3291).

### 3.2. Testing of Driving Waveform Parameters

**Figure 5:**
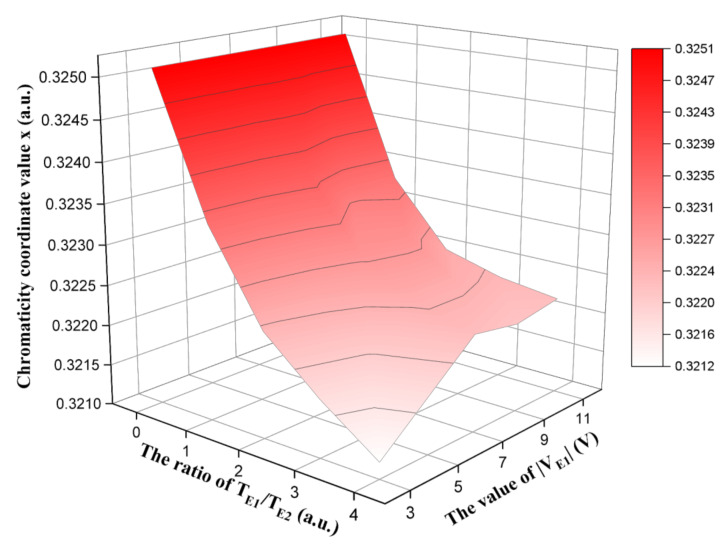
The relationship between x and erasing stage parameters in a white gray scale. The red saturation of the white gray scale reached the highest value when $T_{E1}/T_{E2}$ was 0. The red saturation of the white gray scale reached the lowest value when $V_{E1}$ was −3 V and $T_{E1}/T_{E2}$ was 4.

$V_{E1}$ and $T_{E1}:T_{E2}$ were studied to analyze the effect of these parameters on eliminating red ghost images. $V_{E1}$ was set to −3, −5, −7, −9, and −11 V and $T_{E1}:T_{E2}$ was set to 0:1, 1:1, 2:1, 3:1, and 4:1. The original gray scale of the three-color EPD was set to a black gray scale, and the target gray scale was set to a white gray scale because red ghost images have a greater impact on the gray scale transformation from the black gray scale to the white gray scale. The x of the white gray scale driven by different parameters of the erasing stage is shown in Figure 5. We could observe that the red saturation of the white gray scale reached the highest value when $T_{E1}/T_{E2}$ was 0. It was indicated that red ghost images could not be effectively erased due to the lack of the red erasing stage. The red saturation was decreased with the increase of $T_{E1}/T_{E2}$ when $V_{E1}$ was the same. This phenomenon indicated that the increase of the red erasing stage driving time could increase the distance between red particles and the top of microcapsules, which could reduce the number of residual red particles in the upper part of microcapsules. In addition, the red saturation could not be decreased with the change of $V_{E1}$ when $T_{E1}/T_{E2}$ was less than 2. Instead, the red saturation was decreased with the decrease of $\left|V_{E1}\right|$ when $T_{E1}/T_{E2}$ was greater than 2, and the red saturation reached the lowest value when $V_{E1}$ was −3 V. This phenomenon indicated that a certain driving time of the red erasing stage was necessary to eliminate red ghost images, and the moving speed of red particles could reach the fastest speed when an optimal $V_{E1}$ was determined.

**Figure 6:**
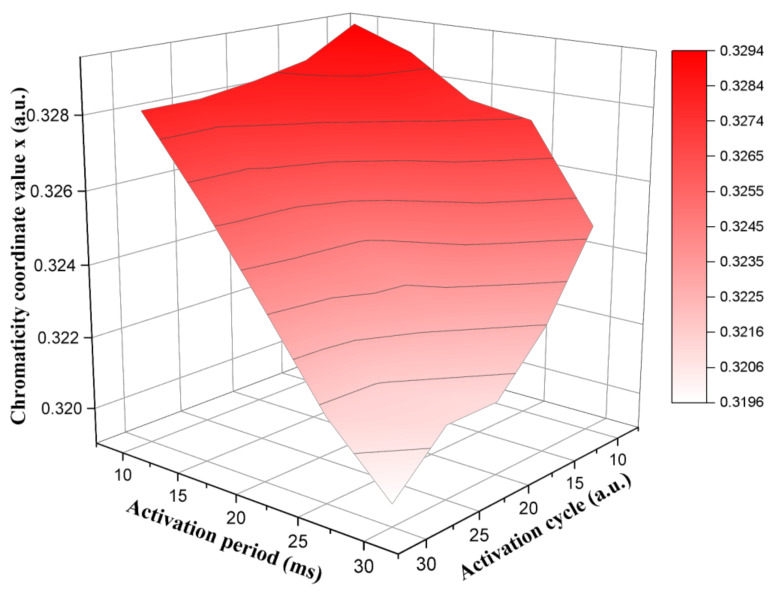
The relationship between x and activation stage parameters in a white gray scale. The red saturation of the white gray scale reached the maximum value when the activation period was 10 ms and the activation cycle was 10. The red saturation of the white gray scale reached the minimum value when the activation period was 30 ms and the activation cycle was 30.

The activation period and cycle of the activation stage were analyzed to achieve an optimal performance of the three-color EPD. The high level voltage and the low level voltage of the activation stage were set to +15 and −15 V, respectively. The activation period was set to 10, 15, 20, 25, and 30 ms. The activation cycle was set to 10, 15, 20, 25, and 30. The x of a white gray scale driven by different activation periods and activation cycles is shown in Figure 6. We observed that the red saturation reached the minimum value when the activation period was 30 ms and the activation cycle was 30. Instead, the red saturation reached the maximum value when the activation period was 10 ms and the activation cycle was 10. Specifically, the red saturation had a negative correlation with activation cycle when the activation period was the same. This indicated that the activity of particles was increased with the increase of the activation cycle. Then, the distance between white particles and the top of microcapsules was decreased, which could increase the luminance of the white gray scale. In addition, the red saturation also had a negative correlation with the activation period when the activation cycle was the same. The increase of the activation period could increase the moving speed of white particles, and the moving speed of red particles was much slower than that of white particles. Then, the mixing probability between white particles and red particles could be decreased.

### 3.3. Performance Comparison

**Figure 7:**
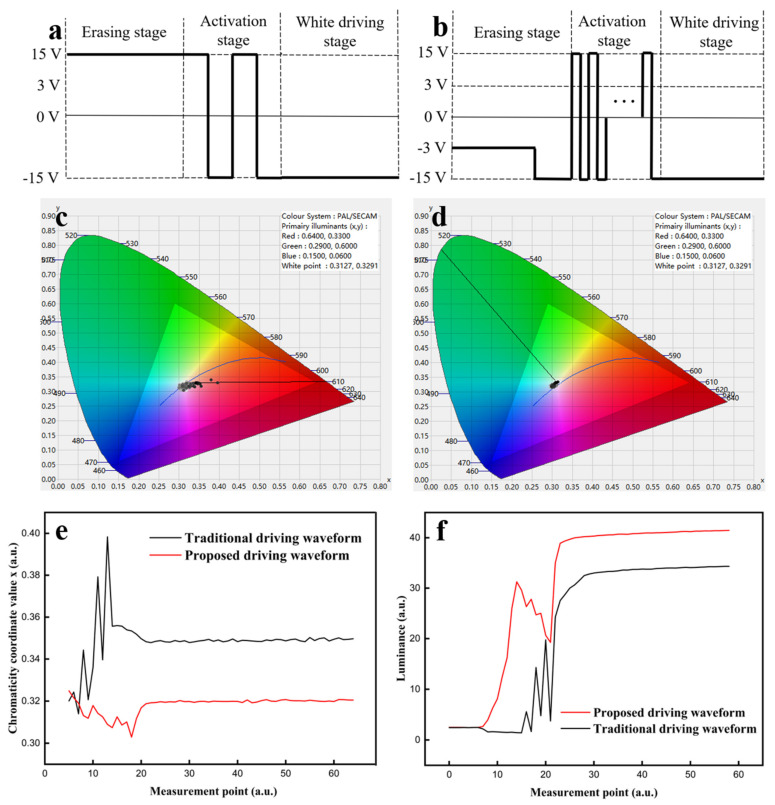
Red saturation comparison between the traditional driving waveform and the proposed driving waveform. (**a**) The traditional driving waveform for forming white gray scale. (**b**) The proposed driving waveform for forming white gray scale. Dots indicate that there are multiple square waves in the activation stage. (**c**) CIE Yxy chromaticity diagram of the traditional driving waveform. (**d**) CIE Yxy chromaticity diagram of the proposed driving waveform. (**e**) x curve comparison between the traditional driving waveform and the proposed driving waveform. The x of the white gray scale formed by the traditional driving waveform was 0.40, which was 0.08 higher than that of proposed driving waveform. (**f**) Luminance curve comparison between the traditional driving waveform and the proposed driving waveform. The luminance of the white gray scale driven by the proposed driving waveform was 41.46, which was 7.14 higher than that of the traditional driving waveform.

A traditional driving waveform [14] was used to compare with the proposed driving waveform, as shown in Figure 7a,b. The luminance and the red saturation of the three-color EPD were analyzed during the gray scale transformation from a black gray scale to a white gray scale. In the traditional driving waveform, the erasing stage driving time was 700 ms, the activation period of the activation stage was 200 ms, the activation cycle of the activation stage was 4, and the black or white driving stage driving time was 700 ms. In the proposed driving waveform, the ratio between the red erasing stage and the original erasing stage was 4:1, the driving voltage of the red erasing stage was 3 V, the activation period of the activation stage was 30 ms, the activation cycle of the activation stage was 30, and the driving time of the black or white driving stage was 280 ms.

The CIE Yxy chromaticity diagrams of the three-color EPD driven by the traditional driving waveform and the proposed driving waveform are shown in Figure 7c,d, respectively. The x curves of the traditional driving waveform and the proposed driving waveform are shown in Figure 7e. We could see that the red saturation of the traditional driving waveform had an oscillating upward trend, and the maximum value of x was 0.40. This was because the traditional driving waveform could not erase the red images, and more and more red particles could remain in the upper part of the microcapsule when the activation stage was applied. Then, the remaining red particles affected the accuracy of the white gray scale, and the x of the white gray scale was 0.35. Instead, the red saturation of the proposed driving waveform had a downward trend. This indicated that red ghost images could be effectively eliminated by the red erasing stage and the activation stage. The x of the white gray scale was 0.32. Therefore, the red ghost images of the white gray scale formed by the proposed driving waveform could be reduced by 80.43%, compared with that of the traditional driving waveform.

Luminance curves driven by the traditional driving waveform and the proposed driving waveform are shown in Figure 7f. We could see that the luminance driven by the traditional driving waveform was decreased at first from the measurement point 6 to 21, which corresponded to the voltage change in the erasing stage and the activation stage. Then, the luminance oscillated, and flickering occurred. The maximum flicker intensity was 16. In contrast, the luminance driven by the proposed driving waveform rose to 31.26 at first. Then, the luminance decreased to 19.24 since the activity of black particles was higher than that of white particles in the activation stage, and the maximum flicker intensity was 3.26. Finally, the luminance of the white gray scale driven by the proposed driving waveform was 41.46, whereas the luminance of the white gray scale driven by the traditional driving waveform was 34.32. This was because red particles and white particles were mixed when the white gray scale was formed by the traditional driving waveform, which reduced the accuracy of the gray scale display. Therefore, the proposed driving waveform could reduce the flicker intensity by 79.63% and increase the luminance of the white gray scale by 20.8%, compared with the traditional driving waveform.

**Figure 8:**
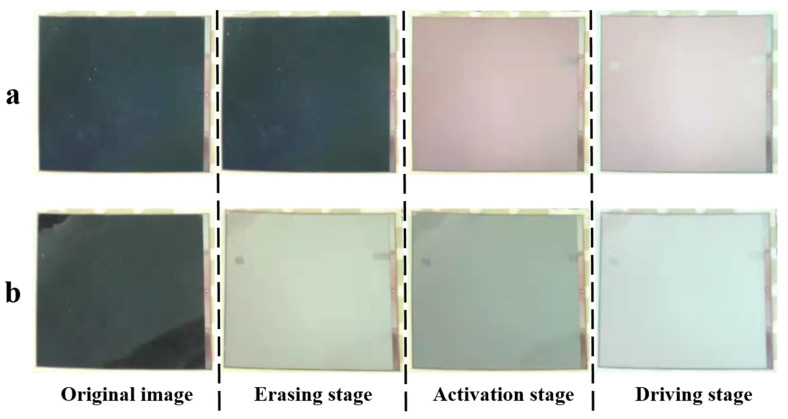
Gray scale transformation comparison between the traditional driving waveform and the proposed driving waveform. (**a**) The gray scale transformation process of the traditional driving waveform. Red ghost images are clearly shown in the white gray scale. (**b**) The gray scale transformation process of the proposed driving waveform. Red ghost images did not appear in the gray scale transformation.

The gray scale transformation process of the traditional driving waveform is shown in Figure 8a. It can be seen that red ghost images appeared in the activation stage, which was consistent with the black line in Figure 7e. Then, red ghost images were clearly shown in the white gray scale. The gray scale transformation process of the three-color EPD driven by the proposed driving waveform is shown in Figure 8b. It can be seen that red ghost images did not appear, which was consistent with the red line in Figure 7e. Meanwhile, the white gray scale could be displayed accurately. In summary, the proposed driving waveform could effectively eliminate red ghost images and improve the accuracy of gray scale display.

## 4. Conclusions

In this paper, we proposed a new driving method based on optimizing the erasing and activation stages for three-color EPDs. In the driving waveform, a new red erasing stage was added for erasing red particles. Red images could be effectively erased by optimizing the parameters of the red erasing stage. Then, the accuracy of the gray scale display could be increased by optimizing the parameters of the activation stage. Finally, the flicker intensity could be decreased by 79.63%, and red ghost images were eliminated effectively by 80.43%. In summary, we designed a driving method for three-color EPDs, which could effectively eliminate red ghost images compared with existing driving methods. This driving method design could promote the development of driving theory and systems for the next generation EPDs.
